# Isolation and characterization of *Ulva prolifera actin1* gene and function verification of the 5′ flanking region as a strong promoter

**DOI:** 10.1080/21655979.2017.1325041

**Published:** 2017-05-19

**Authors:** Chunhui Wu, Peng Jiang, Yang Guo, Jianguo Liu, Jin Zhao, Huihui Fu

**Affiliations:** a Key Laboratory of Experimental Marine Biology, Institute of Oceanology, Chinese Academy of Sciences, Qingdao, China; b College of Earth Sciences, University of Chinese Academy of Sciences, Beijing, China; c Laboratory for Marine Biology and Biotechnology, Qingdao National Laboratory for Marine Science and Technology, Qingdao, China

**Keywords:** *actin*, endogenous promoter, gene structure, leader intron, quantitative GUS assay, *Ulva prolifera*

## Abstract

*Ulva prolifera* is a green macroalgae with an extremely high growth rate that can accumulate biomass with considerable protein content. To set up an available seaweed expression system, a prior step is to isolate endogenous and strong constitutive promoters. For this reason, the full-length genomic *actin1* gene from *U. prolifera* (*Upactin1*) was cloned and its 5′ flanking sequence was obtained by genome walking. The *Upactin1* open reading frame consisted of 1134 nucleotides encoding 377 amino acid residues. Besides 4 exons and 3 introns in the coding region, an extra leader intron was identified in the 5′ untranslated region. According to quantitative GUS assays based on transient expression, the promoter activity of the *Upactin1* 5′ flanking region was found to be several times higher than that of the widely-used cauliflower mosaic virus 35S (CaMV35S) in all tested species of *Ulva*. In addition, precise deletion of the leader intron led to a significant decrease of promoter strength in *U. prolifera*, and almost entire loss of strength in *U. linza* and *U. pertusa*. To our knowledge, this is the first report to prove function of a leader intron in algae. The 5′ flanking region of *Upactin1* was shown to be a much stronger promoter than the foreign CaMV35S, and its activity was highly dependent on the presence of the leader intron. We propose that the *Upactin1* promoter could serve as an endogenous and strong constitutive element for genetic engineering of *U. prolifera*.

## Introduction

*Ulva prolifera* (Ulvaceae, Chlorophyta) is a cosmopolitan species of green macroalgae. It is cultivated for traditional food and has been proposed as a promising resource for valuable compounds or new pharmaceuticals.^1-3^ This macroalgae usually lives in intertidal zones by attaching to substrates with holdfast. In a floating and drifting state, this species can cause and dominate huge green tides by uncontrollable proliferation.^4^ Due to its extremely fast growth rate^5^ and higher protein content,^6^ *U. prolifera* has a greater potential than other seaweeds to become a marine bioreactor that can produce recombinant protein or high-value products in an effective way by metabolic genetic engineering. Besides, unlike kelp and nori for which some specific stages of their life history needed to be conducted in large-scale open-sea areas, *U. prolifera* is very suitable for enclosed cultivation. For example, Hiraoka and Oka^7^ and Carl et al.^8^ have reported a land-based and totally enclosed tank cultivation system for *Ulva*. Therefore, we believe that risk control measures for its environmental release could be easily managed, making *U. prolifera* a potentially outstanding transgenic bioreactor.

Generally, an endogenous and strong constitutive promoter is essential for a stable expression system because it may help positive transformant survival under lethal selective pressure by supplying sufficient resistance, and also contribute to great enhancement of recombinant protein expression. However, because knowledge on seaweed genetics is poor, foreign promoters like simian virus 40 and cauliflower mosaic virus 35S (CaMV35S) have been widely used for a long time.^9^ Recently, promoters of seaweed genes such as *GAPDH, tubulin, actin*, and *rbcS* have been isolated and applied to seaweed genetic transformations.^10-13^ In *Ulva* species, the CaMV35S promoter could only drive weak transient expression in *U. lactuca*,^14^ while the endogenous *rbcS* promoter, which was probably induced by the photoperiod,^12^ helped to obtain stable transformants in *U. mutabilis*.^15^ Therefore, to set up an applicable expression system in *U. prolifera*, it is a prior step to isolate strong constitutive promoters of *U. prolifera* genes.

ACTIN is an essential component of the cytoskeleton in eukaryotic cells, where it plays fundamental roles in, for example, cytoplasmic streaming, cell division, cell shape determination, organelle movement, and endocytosis.^16^ In plants, the *actin* 5′ flanking region has been proved widely as a constitutive promoter with much stronger activity than that of the exogenous CaMV35S promoter.^17-21^ In addition, some elements in the *actin* promoter, like a leader intron in the 5′ untranslated region (UTR), have been identified,^22,23^ and their vital functions have been clarified by quantitative analysis based on transient expression.^17,18,21^ In algae, *actin* genes have been found in most representative taxon including green, red, and brown algae, and diatoms.^24-27^ Although several *actin* promoters have been isolated in seaweed, except for the promoter from *Pyropia yezoensis*, none have been applied.^13^ Moreover, the leader intron of algal *actin* genes has been detected only in green macro- and microalgae,^25,27,28^ but its function is still unknown.

In this study, we aimed to amplify the *actin* gene of *U. prolifera* using degenerate primers based on homologous sequences from other *Ulva* species, and to obtain its 5′ flanking region to quantitatively determine promoter function using a transient expression system. This work could provide a constitutive promoter for genetic engineering of *U. prolifera*.

## Results

### Characterization of the *actin1* gene from *U. prolifera*

**Figure 1.**
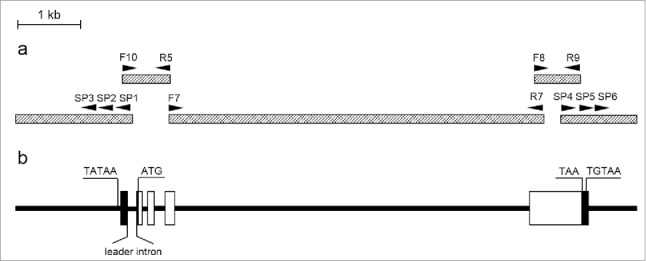
Diagrammatic illustration of the assembly of *Upactin1* and locations of all primers (a), and schematic representation of the *Upactin1* gene structure (b). In (a), solid triangles indicate the primers used for genomic cloning and walking. In (b), the horizontal bars correspond to introns and flanking DNA, white rectangles represent *actin* coding exons, and black rectangles indicate transcribed but untranslated regions of the exons. The relative positions of the putative TATA box (TATAA), translational start (ATG) and stop (TAA) codons, and putative polyadenylation signal (TGTAA) are shown.

The amplified 5′ RACE (rapid amplification of cDNA ends) fragment of the *actin1* gene from *U. prolifera* (*Upactin1*) was 1136 nt in length and contained a 5′ UTR of 129 nt, and the 3′ RACE fragment was 1095 nt in length and contained a 3′ UTR of 127 nt. The full length of the *Upactin1* cDNA sequence, which was assembled with the 5′ and 3′ RACE fragments, was 1390 nt and included the entire open reading frame of 1134 nt encoding 377 amino acids. Because attempts to obtain the entire *Upactin1* genomic sequence using one pair of primers have always failed, instead, we used 3 pairs of specific primers (F10/R5, F7/R7, and F8/R9) to amplify 3 overlapping genomic fragments of 760 nt, 5974 nt, and 724 nt, respectively (Fig. 1a). After genome walking for the 5′ and 3′ flanking regions of *Upactin1*, a 1941 nt fragment upstream of the ATG start codon and a 870 nt fragment downstream of the TAA stop codon were obtained. Finally, we assembled a 9853 nt genomic sequence of *Upactin1* using the 5 overlapping fragments (Fig. 1a).

**Figure 2.**
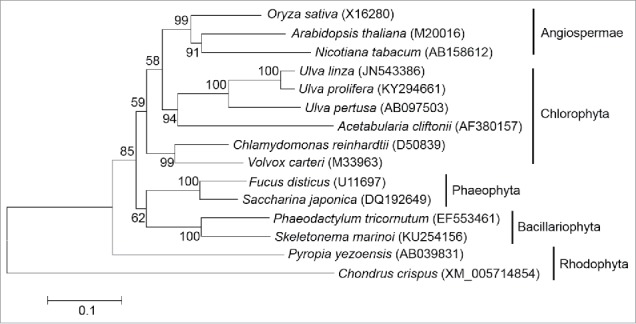
Phylogenetic neighbor-joining tree based on complete *actin* cDNA coding sequences from algae and higher plants.

By comparing the cDNA and assembled genomic sequences, the structure of the *Upactin1* gene was characterized (Fig. 1b). Four exons and 3 introns were identified in the coding region. Except for these 3 introns, which were located after the codons specifying the 20th (Intron2), 52nd (Intron3), and 102nd (Intron4) amino acids (AAs), an additional intron (Intron1 or leader intron) was detected in the 5′ UTR from −172 nt to −27 nt upstream of the ATG start codon. The consensus GT donor and AG acceptor sequences were found in all 4 introns. In the phylogenetic neighbor-joining tree constructed with the complete cDNA coding sequences of *actin* from algae and higher plants, *Upactin1* clustered together with *actin* sequences from other *Ulva* species (Fig. 2). Further, the phylogenetic tree showed that the green macroalgae shared a closer relationship with higher plants than the green microalgae *Chlamydomonas reinhardtii* and *Volvox carteri*.

### Bioinformatic analysis of the *Upactin1* 5′ flanking region

**Figure 3.**
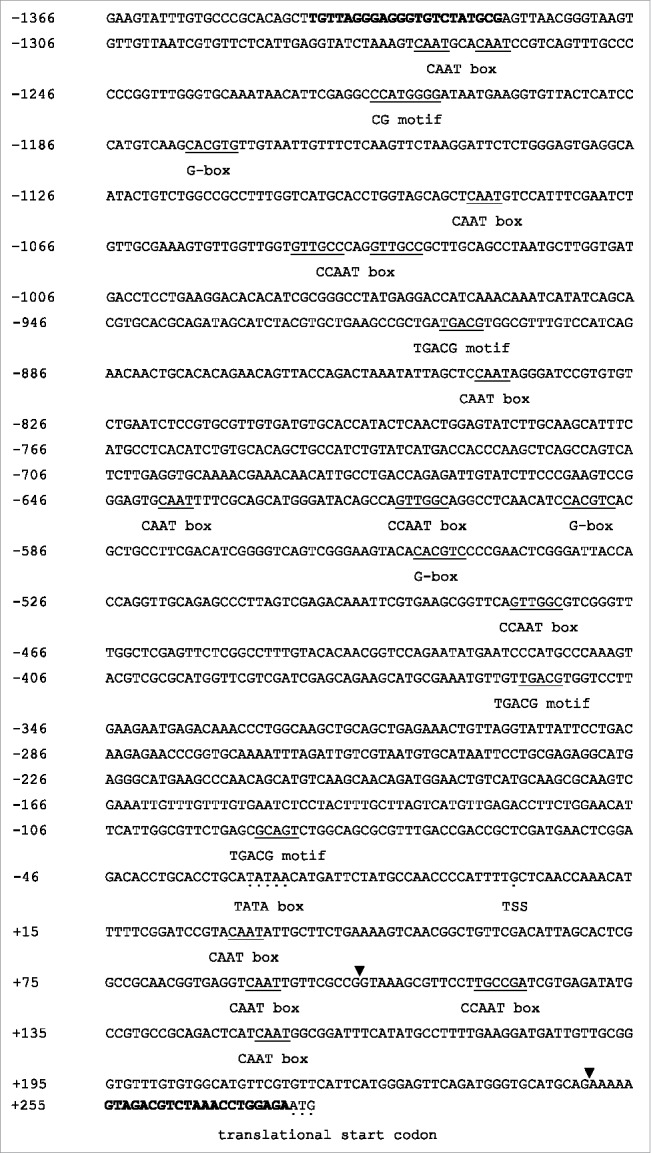
Nucleotide sequence of the *Upactin1* 5′ flanking region and predicted regulatory elements. Numbers in the left margin start from the TSS (+1). The bold nt sequences correspond to the locations of the F13 and R13 primers. Presumptive cis-elements are underlined. The putative TATA box (TATAA), TSS, and translational start codon (ATG) are indicated by dots under the sequence. The splice junction borders of the leader intron are marked by solid triangles

The predicted TATA box and cis-acting regulatory DNA elements in the *Upactin1* 5′ flanking region were identified by the PlantCARE program, as shown in Figure 3. The putative transcription start site (TSS) was located at 275 nt upstream of the ATG start codon. The presumptive TATA box (TATAA) was located from −30 to −26 nt upstream of the TSS. Some regulatory cis-acting elements, such as CAAT box, CCAAT box, G-box (CACGTG), CG motif (CCATGGG), and TGACG motif, were also detected. A putative algal polyadenylation signal (TGTAA) were found 80 nt downstream of the TAA stop codon.^29^ Because almost all the predicted cis-elements were located within a 1.6 kb region upstream of the ATG start codon, we chose this region to construct vectors to verify the promoter activity.

### Vector construction

**Figure 4.**
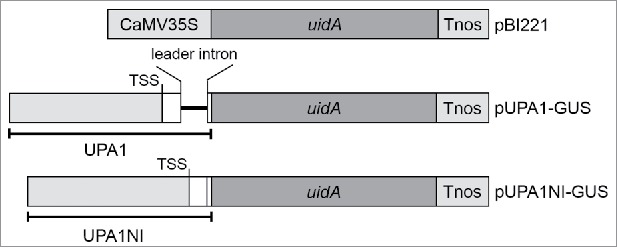
Schematic drawing of the chimeric GUS expression cassettes used for transformations to *Ulva*. Reporter gene *uidA* encodes the GUS protein, Tnos indicates the terminator of the *nos* gene, and TSS is the transcription start site.

To verify the promoter activity of the *Upactin1* 5′ flanking sequence, especially the function of the identified leader intron, 2 vectors, pUPA1-GUS and pUPA1NI-GUS, were constructed using pBI221 (Clontech) as a backbone vector (Fig. 4). Fragment UPA1 was amplified with primers F13 and R13 and its sequence contained a wild-type 1618 nt region upstream of the *Upactin1* ATG start codon that included the leader intron. Fragment UPA1NI was amplified with primer F13 and another 3′ primer, R18, to precisely delete the leader intron. Then, the same pair of adapters was added to both ends of UPA1 and UPA1NI by primers F13B and R13B. After that, vector pBI221 was double digested with the restriction endonucleases *Hin*dIII and *Sma*I to remove the CaMV35S promoter. The remaining promoter-less vector was ligated to UPA1 and UPA1NI by homologous recombination between adapters and ends of the vector using an In-Fusion*®* HD Cloning Kit (Clontech).

### Quantitative analysis of GUS activity

**Figure 5.**
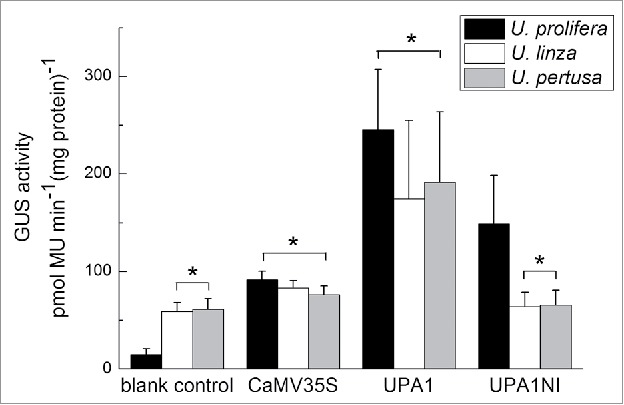
Quantitative GUS activity in blank control and treated groups in 3 species of *Ulva*. Background of GUS activity refers to the values obtained in the blank controls. Significant differences are indicated (*P >0.05).

GUS quantitative analysis showed that the background level in cultured *U. prolifera* was as low as 14.64 pmol MU min^−1^ mg^−1^ protein (Fig. 5). Compared with CaMV35S, for which the GUS activity was 91.44 pmol MU min^−1^ mg^−1^ protein in *U. prolifera*, the UPA1 fragment contained the entire 5′ flanking sequence of *Upactin1* and showed the highest GUS activity of 245.34 pmol MU min^−1^ mg^−1^ protein (P <0.05). The leader intron-deleted UPA1NI fragment showed a significant decrease of GUS activity to 148.96 pmol MU min^−1^ mg^−1^ protein (P <0.05), but was still much higher than the value for CaMV35S (P <0.05). By deducting the GUS background value in all treated groups, we found that UPA1 exhibited GUS activity about 3-fold higher than CaMV35S, while UPA1NI led to a decrease in GUS activity of 42% compared with UPA1.

Unlike *U. prolifera*, which was cultured in the laboratory, the GUS background activity in the 2 wild samples was much higher at about 60 pmol MU min^−1^ mg^−1^ protein. In *U. linza* and *U. pertusa*, the GUS activity by the CaMV35S promoter was low at 83.06 and 76.14 pmol MU min^−1^ mg^−1^ protein, respectively. The UPA1 fragment still showed significantly higher activity than CaMV35S at 174.19 or 191.37 pmol MU min^−1^ mg^−1^ protein. After deducting the GUS background value in all treated groups, we found that the activity of UPA1 was about 4.81 or 8.67 times higher than that of CaMV35S, and while the activity of UPA1NI was partial decreased in *U. prolifera*, almost all its activity was reduced to the GUS background level in both wild species.

## Discussion

We cloned and characterized the full length *Upactin1* gene. The *Upactin1* open reading frame region was 1134 nt encoding 377 AAs, which is the most conserved length for plant *actin* genes. We found all the determined amino acid sequences of ACTIN in *Ulva* were quite conserved. Although the cDNA sequence similarity was 96% between *Upactin1* and *U. linza actin1*, the translated amino acid sequences were identical. *Upactin1* shared a lower similarity of 84% with *U. pertusa actin* cDNA, but only the 321st amino acid was different in the translated amino acid sequence (Thr in *U. prolifera* and Ala in *U. pertusa*).

*Actin* is considered as a highly-conserved housekeeping gene involved in fundamental cellular processes, and the structural features of its introns, such as number, location, and size, are of evolutionary importance. In *Upactin1*, the coding region consisted of 4 exons and 3 introns, preceded by one leader intron in the 5′ UTR region. This scheme is identical to that of *U. pertusa*,^25^ and quite universal in land plants.^22,23,30^ However, this scheme has never been detected in other studied algae where the *actin* genes were always found to be interrupted by different numbers of introns.^24,27,28,31^ In Rhodophyta, only one intron was found in the *actin* coding regions and no leader intron was detected in the 5′ UTRs.^24,31^ In green microalgae *C. reinhardtii* and *V. carteri*, up to 8 introns have been detected in *actin* coding regions. These findings indicate that the *actin* of green macroalgae seems to have a closer relationship with the *actin* of land plants than with green microalgae, which is in line with the cluster features in the phylogenetic tree we constructed using *actin* cDNA coding sequences (Fig. 2).

With respect to intron location in the coding region, Intron2 of *Upactin1* was located after the codon for the 20thaminoacid residue, and this position is quite conserved in animals, land plants, and green algae.^22,23,25,32^ Intron3 was located after the codon for the 52ndaminoacid residue, and this position is conserved in green algae.^25^ Notably, unlike all studied green algae, the position of Intron4 in *Upactin1* was after the codon for the 102nd amino acid residue instead of the 101st. Besides the location of introns in the coding region of *actin* genes, the sequence length between leader intron and ATG in the 5′ UTR also varies among species. It is 10 nt in dicots, monocots and moss species,^17^ 8 nt or 10 nt in green microalgae,^27,28^ and we found it was 27 nt in *U. prolifera*, the same as in *U. pertusa*.^25^ In addition, the size of *actin* leader introns from higher plants was usually larger than that of introns in coding regions.^30^ However, in both *U. pertusa* and *U. prolifera*, the leader intron (156 or 146 nt) was much shorter than the longest intron in the coding regions (2067 or 5671 nt).

According to the quantitative measurement of GUS activity, the *Upactin1* 5′ flanking region was confirmed to function as a strong promoter in *U. prolifera*. Compared with CaMV35S, the most widely used exogenous promoter in plants, the GUS activity driven by the endogenous entire UPA1 fragment was about 3-fold higher. Such significant enhancement (from 2- to 3-fold) has been documented repeatedly in plants,^17,18^ and in certain extreme situations, an 11-fold increase was obtained due to the very low strength of CaMV35S.^21^ Moreover, *actin* promoters from plants usually show universal functions among different species.^17,33^ In the *Ulva* genus, 3 thalli forms are known to exist. *U. prolifera* and *U. pertusa* represent single-layer tubular and 2-layer blade thalli, respectively. *U. linza* is a transitional form. In our experiment, the activity of the entire *Upactin1* 5′ flanking region in these 3 forms of *Ulva* was similarly high and much higher than that of CaMV35S. This finding suggested that the *Upactin1* 5′ flanking region could probably serve as a universal promoter in the *Ulva* genus.

Our experiment clearly demonstrated that the leader intron in the 5′ UTR played a crucial role in the strength of the *Upactin1* promoter. In *U. prolifera*, complete deletion of the leader intron led to a 42% reduction of GUS activity, and the value dropped to the background level in *U. pertusa* and *U. linza*. To our knowledge, this is the first report to prove the function of the leader intron in algae. In land plants, similar effects have been revealed by deletion of leader intron, which resulted in significant decreases of *actin* promoter activity.^17,19,20^ In addition, insertion of a leader intron into the CaMV35S promoter enhanced its strength in some cases.^21^ The leader intron has been found universally in *actin* genes from most animals, land plants, and green algae,,^22,23,25,32^ and it has been detected more widely in close to 20% of the surveyed genes in *Arabidopsis thaliana*.^34^ Two main hypotheses have been suggested to explain the regulatory mechanism of the leader intron; one is presence of a potential enhancer within its region,^20^ and the other is its special splicing process.^17,19^

Due to weak endogenous GUS activity in plants and its wide distribution in microbes, *uidA* has been the most popular reporter gene used in plants coupled with a strict sterilization process.^35^ We detected a low GUS background in the blank control of *U. prolifera*, which originated from a single cell that was cultured in the laboratory, but unignorable values in freshly collected *U. pertusa* and *U. linza*. The fact that the surfaces of *Ulva* are usually fully attached by epiphytic bacteria in their natural habitat,^36^ may explain the origin of the elevated GUS background detected for the wild samples in our experiment. Despite this, the GUS background did not interfere with the conclusion that the promoter activity of the *Upactin1* 5′ flanking region was much higher than that of CaMV35S in all treated groups.

In conclusion, we cloned the full length of the *Upactin1* gene and characterized its structure. The 1.6 kb 5′ flanking region of *Upactin1* has been proved to function as a much stronger promoter than CaMV35S, and it function was highly dependent on the existence of a leader intron. For genetic engineering of *U. prolifera*, an endogenous *Upactin1* promoter could serve as a constitutive regulatory element, and its leader intron should to be inserted into other promoters to test its effect on activity enhancement. In addition, because *actin* genes play important roles in cell growth and division, the characterization of the *Upactin1* gene and its 5′ regulatory region may help in analyzing the mechanism of green tide in the Yellow Sea. *Upactin1* also could serve as an appropriate reference gene and internal control in RT-qPCR assays, which would facilitate the expression analysis of genes corresponding to *Ulva* blooms.

## Materials and methods

### Sample culture and species identification

Attached *U. prolifera* were collected in July 2011 from the coastal area of Jiangsu Province, China (34°30′ N, 119°45′ E). Samples were washed with cold boiled seawater for 3–5 times and then stored in Von Stosch's Enriched (VSE) medium. Individuals were selected for unialgal and clonal culture with a 12:12 h light:dark cycle under 50 μmol photons m^−2^ s^−1^ irradiance at 17°C.^37^ In literature, the optimum temperature range for vegetative growth was 15–25°C. However, in our samples, the thalli tended to release zoids when cultivated at temperatures above 20°C, while the growth rate was relatively lower when cultivated below 15°C. Therefore, we chose 17°C for relative high growth rate. Attached samples of *U. linza* and *U. pertusa* were collected in January 2016 from Huiquan Bay, Qingdao, China (36°3′ N, 122°20′ E). These 2 samples were rinsed in sterilized cold boiled seawater to remove debris and epiphytes, and then used immediately for genetic transformation. Species identification for all the *Ulva* samples was performed using rDNA internal transcribed spacer (ITS) as a molecular genetic marker.^38^

### cDNA cloning and RACE of *Upactin1*

Total RNA extraction from fresh thalli of *U. prolifera* was performed using an *EasyPure®* Plant RNA Kit (Transgen, China). The concentration and quality of the total RNA was determined by UV absorbance and agarose gel electrophoresis, respectively. Single-strand cDNA was synthesized using a M-MLV First Strand Kit (Invitrogen). Based on the cDNA sequence of *actin1* in *U. linza* (GenBank Accession No. JN543386) and *actin* in *U. pertusa* (AB097503), a pair of degenerate primers, F1 and R1, (all primers sequences are shown in Table 1) was designed to amplify *Upactin1* cDNA fragments. A polymerase chain reaction (PCR) was performed at 94°C for 5 min, followed by 30 cycles of 94°C for 50 s, 53°C for 50 s, 72°C for 90 s, and a final extension at 72°C for 10 min. The obtained cDNA sequence was used to design specific primers, NGSP1 and GSP2, for 5′ and 3′ RACE, respectively, which were performed using a SMARTer*®* RACE 5′/3′ Kit (Clontech) according to the manufacturer's instruction. All PCR products were cloned into pGEM*®*-T Easy vector (Promega) and then sequenced at the Sangon Biotech Company. The *Upactin1* cDNA coding sequence was aligned with homologous sequences from representative taxon of algae and higher plants using Clustal X 1.83. The aligned sequences were used to construct a phylogenetic neighbor-joining tree using MEGA 5.0 according to the Kimura 2-parameter model with a bootstrap value of 1000.

**Table 1.** All the primers used in this research

Amplification	Primers	Sequence (5′-3′)
cDNA and genomic cloning	F1	ATGGGAGACGAAGGMGAGGT
	R1	YTAAAARCACTTCCTGTG
	R5	GGTGCCAGATCTTCTCCAT
	F7	CGGCATTGTAACAGATTGG
	R7	GTGGCATAGCATAACCTTCATA
	F8 R9	TCAAGCGGTTCTGTCGTT GGGTTAAAAACACTTCCTGTG
	F10	GATCCGTACAATATTGCTTC
vectors construction	F13	TGTTAGGGAGGGTGTCTATGCG
	R13	TCTCCAGGTTTAGACGTCTAC
	R18	TCTCCAGGTTTAGACGTCTACTTTTTCGGCGAACAATTGACCTCACCG
	F13B	tgattacgccaagcttTGTTAGGGAGGGTGTCTATGCG^a,b^
	R13B	agggactgaccacccgggTCTCCAGGTTTAGACGTCTAC
5′ and 3′ RACE	NGSP1	GATTACGCCAAGCTTACGGAGAACTTACGCTCTGGTGGC
	GSP2	GATTACGCCAAGCTTTTCGGCTGTCGTCTGCGATAATGG
genome walking	SP1	CAAGGCAATCAGTAATGGACACG
	SP2 SP3	GTGCCGAGGTCTGCCAACGATT CCCGCAACAATCATCCTTCAAAA
	SP4	GATTGTGGTGACGGTGTTAGCC
	SP5	TTTACGACTTCAGCGGAACGAG
	SP6	GGATTACGAACAGGAGATGGCTAC

a Lowercase letters indicate adapters that are homologous to the end of the promoter-less pBI221 vector used for ligation by recombination.

b Underlined letters indicate recognition sites of *Hin*dIII (AAGCTT) and *Sma*I (CCCGGG).

### Genomic cloning of *Upactin1* and its flanking region

Total genomic DNA of *U. prolifera* was extracted using a Plant Genomic DNA Kit (Tiangen). Based on the *Upactin1* cDNA sequence, 3 pairs of specific primers (F10/R5, F7/R7, and F8/R9) were designed to amplify the corresponding genomic fragments. The overlapping regions between these 3 fragments were used to assemble the full length *Upactin1* genomic sequence. To obtain the flanking region at both ends, 6 specific primers (SP1, SP2, and SP3 for the 5′ flanking region, and SP4, SP5, and SP6 for the 3′ flanking region) were designed to perform genome walking using a Genome Walking Kit (TaKaRa). The TATA box and cis-elements were predicted using the online sequence analysis program PlantCARE (http://bioinformatics.psb.ugent.be/webtools/plantcare/html/).^39^

### Transformation to *Ulva*

For genetic transformation, a Biolistic PDS-1000/He Particle Delivery System (Bio-Rad) was used. Before bombardment, tubular thalli of *U. prolifera* and *U. linza* were cut into short segments less than 2 cm long, and sheet thalli of *U. pertusa* were cut into circular discs less than 2 cm in diameter. Then, pieces of seaweed were placed on a circular sterilized filter paper and gathered in the central area. According to the operation protocol described for seaweed,^40^ vectors were adhered onto gold particles (0.6 μm in diameter). For each bombardment, the same parameters, including 1.0 μg DNA, 1100 psi of rupture pressure, and 6 cm of particle travel distance, were followed. Three independent experiments (biologic replicates) were performed. After transformation, the bombarded thalli were allowed to recover for 8 h in dark, then cultured under normal conditions as described above. Untransformed thalli were used as blank controls.

### Quantitative analysis of GUS activity

Protein crude extracts were prepared 48 h after transformation. For each group, approximately 100 mg thalli were homogenized with liquid nitrogen, then 1 mL GUS extraction buffer (50 mM Na_2_HPO_4_, 10 mM Na_2_EDTA, 0.1% Triton X-100, and 10 mM β-mercaptoethanol) was added. The homogenates were centrifuged at 10,000 × g for 15 min at 4°C, then the supernatants were transferred to a new tube and stored on ice for further use. The soluble protein concentration was determined by a Bradford Protein Assay Kit (Tiangen).

GUS activity was quantified by fluorometric 4-methylumbelliferyl-β-D-glucuronide (4-MUG) assay, as described by Martin.^41^ Each GUS reaction solution consisted of 60 μL prepared protein crude extracts and 540 μL 37°C pre-warmed assay buffer (50 mM Na_2_HPO_4_, 10 mM Na_2_EDTA, 0.1% Triton X-100, 10 mM β-mercaptoethanol, and 2 mM 4-MUG). The reaction solutions were incubated at 37°C for 60 min. At both start (0 min) and stop points (60 min), each 100 μL of reaction solution was transferred to 900 μL stop buffer (0.2 M Na_2_CO_3_). The fluorescence intensities were determined with a microplate-reader (Infinite M1000 PRO, TECAN) at excitation wavelength of 365 nm and emission wavelength of 455 nm. Each sample was detected in triplicate and then analyzed by t-test and one-way ANOVA. Differences were considered statistically significant at P <0.05. Statistical analysis was performed using the statistical software SPSS 18.0.
